# CAD/CAM Abutments in the Esthetic Zone: A Systematic Review and Meta-Analysis of Soft Tissue Stability

**DOI:** 10.3390/jcm12113847

**Published:** 2023-06-04

**Authors:** Diego Lops, Eugenio Romeo, Magda Mensi, Giuseppe Troiano, Khrystyna Zhurakivska, Massimo Del Fabbro, Antonino Palazzolo

**Affiliations:** 1 Department of Biomedical, Surgical and Dental Sciences, Dental Clinic, School of Dentistry, University of Milan, 20122 Milan, Italy; diego.lops@unimi.it (D.L.);; 2 Department of Surgical Specialties, Radiological Science and Public Health, University of Brescia, 25123 Brescia, Italy; 3 Department of Clinical and Experimental Medicine, University of Foggia, 71122 Foggia, Italy; 4 Department of Biomedical, Surgical and Dental Sciences, University of Milan, 20122 Milan, Italy; 5 Fondazione IRCCS Ca’ Granda Ospedale Maggiore Policlinico, 20122 Milan, Italy

**Keywords:** computer-aided design, computer-aided manufacturing, dental implants, esthetic zone, soft tissue, titanium abutments, zirconia abutments

## Abstract

Computer-aided design and computer-aided manufacturing customized abutments are increasingly used in everyday clinical practice. Nevertheless, solid scientific evidence is currently lacking regarding their potential advantages in terms of soft tissue stability. The main aim of this systematic review and meta-analysis was to compare the soft tissue outcomes of prefabricated versus customized (CAD/CAM) abutments. The present review was registered with PROSPERO (CRD42020161875) and the protocol was developed according to the PRISMA statement. An electronic search was performed on three databases (PubMed, Embase and Cochrane Central) up to May 2023. Data extraction was followed by qualitative and quantitative analysis of the included studies. Three randomized controlled clinical trials and three controlled clinical trials (number of patients = 230; number of dental implants = 230) with a follow-up of between 12 and 36 months were included. No significant differences were observed between prefabricated versus customized (CAD/CAM) abutments regarding midfacial mucosal recession, interproximal papillae and pink aesthetic score (PES) after 12 months. Conclusion: The potential benefits of CAD/CAM abutments on soft tissues should be better clarified in future investigations. The usage of customized CAD/CAM abutments in everyday clinical practice should be based on a careful case-by-case evaluation (CRD42020161875).

## 1. Introduction

Nowadays, the replacement of missing teeth in the aesthetic area through dental implants is considered a safe and predictable treatment modality [1]. Nevertheless, increasing aesthetic expectations need to be considered, especially concerning white and pink aesthetics [2]. On the one hand, white aesthetics is related to the type and characteristics of ceramics for abutments, frameworks and veneering. On the other hand, soft tissue integration depends on several aspects, such as: the initial peri-implant mucosal phenotype, surgical procedures (e.g., soft tissue grafting and submerged or nonsubmerged implants) and implant features, such as the timing of placement and loading [3]. Furthermore, pink aesthetics might be hindered in the case of high smile lines, thin buccal hard and soft tissues, scars as a consequence of previous interventions and toothbrushing trauma. Hence, the integration of implant-supported restorations with the neighboring teeth and soft tissues is of utmost importance for the final success of the treatment [4,5].

A key role in long-term success is played by the stability of the buccal soft tissue margin and the presence of a complete interproximal papilla [6]. Although it has been reported that minor soft tissue recessions and asymmetries tend to be neglected, it should be considered that exposure of the metallic portion might seriously impair patient satisfaction and, as a consequence, the overall treatment success [7,8]). At the same time, the entire filling of the papilla under the contact point is a particularly challenging condition. In fact, interproximal papilla loss leads to so-called black triangle syndrome which is characterized by cosmetic impairment, uncomfortable phonetics and lateral food impaction [9]. It has been reported that a vertical distance < 5 mm from the bone peak and the prosthetic contact point, and a horizontal distance > 1.5 mm between the dental implant and the adjacent tooth, are significantly linked to the presence of the interproximal papilla in the case of implants with platform-switching abutment connection [10,11]. Thereafter, several authors have investigated the role of such dental implant features, claiming that a high percentage of midfacial and interproximal soft tissue recessions could be prevented [12,13]. Hence, in modern implant dentistry, the correct choice of dental implants and components is crucial to obtain a pleasant esthetic outcome with long-term success. The emerging literature shows the relationships between abutment type and shape and better soft tissue integration. Abutment geometries allowing more space for soft tissue promote the formation of the so-called “O-ring”, which is a circumferential arrangement of connective tissue surrounding the implant that could reduce soft tissue shrinkage [14]. In the beginning, implant abutments were only available as prefabricated stock parts that were provided by manufacturers. However, since the early 1990s, computer-assisted design/computer-assisted manufacturing (CAD/CAM) systems have progressively been introduced in abutment production in order to compensate for poor implant placement, optimize the emergence profile and improve peri-implant mucosal support. In fact, using CAD/CAM technology, clinicians can individualize the shape and tilt of abutments, thus modulating the compression, support and space for peri-implant soft tissues [15]. Moreover, CAD/CAM technology promotes ceramic abutment customization. In the beginning, ceramic abutments were available only as alumina or zirconia abutments. Due to its better mechanical properties, zirconia has overtaken alumina as the preferred ceramic abutment material [16]. One more advantage is the possibility to carefully choose the location of the cementation margin so as to enhance remnant removal. Nonetheless, despite the wide spread and good clinical results, currently, weak scientific evidence is available on this topic. As a consequence, the role of the customization of implant abutments in aesthetic outcomes should be further elucidated [17]. Numerous systematic reviews and meta-analyses have previously investigated the role of CAD/CAM abutments on pink esthetic score (PES) [6,18,19]. Unfortunately, no clinical data on interproximal soft tissues (e.g., peri-implant papillae) and vestibular soft tissue margin (e.g., midfacial mucosal recession) have been provided. To the best of our knowledge, this is the first systematic review and meta-analysis investigating the effects of customized CAD/CAM abutments on peri-implant soft tissues. Accordingly, the primary aim was to investigate whether CAD/CAM abutments could improve soft tissue outcomes in single-tooth rehabilitation when compared to prefabricated (stock) abutment. Soft tissue-related parameters such as midfacial mucosal recession (ML), interproximal papilla recession (IntPapilla) and pink esthetic score (Overall PES) were evaluated after at least 12 months of loading. 

## 2. Materials and Methods

The protocol of the present systematic review was developed according to the “Preferred Reporting Items for Systematic Reviews and Meta-Analyses” (PRISMA) guidelines and the Cochrane Handbook. Furthermore, it was registered in the International Prospective Register of Systematic Reviews (PROSPERO, www.prospero.org, accessed on 11 December 2019) with ID: CRD42020161875. 

### 2.1. PICOS

The review was performed in order to answer the following PICOS question: “In patients requiring a single implant in the esthetic zone, does the presence of CAD/CAM abutment improve soft tissue stability after at least 12 months of loading?” 

The present question was accomplished via the following method:

P = Patients affected by partial edentulism restored with single implant crown in the aesthetic zone (incisors, canines or premolars) placed in maxilla or both jaws.

I = Prosthetic rehabilitation through customized (CAD/CAM) abutment.

C = Prosthetic rehabilitation through prefabricated (stock) abutment.

O = Pink esthetic score (PES) or numeric measurements in millimeters.

S = Randomized controlled clinical trials (RCTs) or controlled clinical trials (CCTs) with at least 12 months of follow-up.

### 2.2. Inclusion Criteria 

Only prospective interventional studies with a control group evaluating single implant placement in the esthetic zone, meaning the maxillary or mandibular segment between and including the second premolars, were included. Moreover, they were considered eligible if they presented with at least 12 months of follow-up after implant loading and 10 patients.

### 2.3. Exclusion Criteria 

Human prospective studies missing the inclusion criteria were excluded from the review, as were in vitro studies, animal studies, retrospective studies, observational studies, case reports and narrative or systematic reviews.

### 2.4. Search Strategy

The search strategy exclusively considered human studies in the English language published up to and including the 15 May 2023. 

The online search was accomplished using MEDLINE (PubMed), EMBASE and the Cochrane Central Register of Controlled Trials (CENTRAL). Literature searching was independently conducted in an unblinded manner by 2 reviewers (AP & DL). The bibliographic search consisted of a combination of MeSH terms and free-text words combined through Boolean Operators (AND or OR). An example of the words used for the search process (PubMed) is as follows: (anterior OR esthetic) AND (abutment OR dental implant OR zirconia OR titanium abutment OR CAD/CAM abutment OR custom abutment) AND (gingival margin OR papilla OR soft tissue stability OR gingival recession). The search strings used in each database are listed in Supplementary Table S1. A partial search of the gray literature was performed on www.opengray.eu. Moreover, hand searching for potentially missed articles was performed on *Clinical Implant Dentistry and Related Research*, the *Journal of Oral and Maxillofacial Implants*, *Clinical Oral Implants Research*, *Implant Dentistry*, the *Journal of Dental Research*, the *Journal of Clinical Periodontology*, the *Journal of Periodontology*, the *International Journal of Periodontics and Restorative Dentistry*, the *Journal of oral rehabilitation*, and the *International journal of prosthodontics* from January 2010 up to May 2023. These journals were selected since they were deemed more relevant to peri-implant soft tissues. Other prolific journals, such as *Gerodontology* and *Dental Materials*, were not investigated. Additionally, a search in the bibliographies of the included articles was conducted by the two reviewers. The whole search process was conducted via examiner calibration, and calibration consisted of two rounds in which the reviewers independently assessed the validity of 40 titles and articles from the search for their inclusion in the following step. At the end, the level of agreement was calculated (k = 0.88). Any disagreement on the eligibility of the included studies was resolved through discussion.

### 2.5. Data Extraction

Data of the included articles were retrieved on the basis of an ad hoc extraction sheet by two reviewer authors (AP & DL). Any controversy was resolved through discussion with a third reviewer not directly involved in the search process (MM). The following data were tabulated from the studies meeting the inclusion criteria: author names and study design, year of publication, follow-up, abutment material, abutment type, interdental papilla, interproximal gingival recession, marginal soft tissue stability, implant type, implant position, regenerative procedures, implant and prosthetic survival rate, complications, sample characteristics (number of patients, gender and mean age/range). All extracted data are summarized in Table 1. Corresponding authors were contacted in cases of uncertainty on the quality assessment section and/or missing results.

### 2.6. Quality Assessment

In the case of the non-randomized clinical trial (non-RCT), the quality assessment of the included studies was performed through the tool “Risk of Bias In Non-randomised Studies of Intervention” (ROBINS-I: a tool for assessing risk of bias in non-randomized studies of interventions; BMJ, 2016). The tool was designed so as to determine the risk of bias pre-intervention, intervention and post-intervention, and is composed of seven items: bias due to confounding, bias in selection of participants for the study, bias in the classification of interventions, bias due to deviations from the intended interventions, bias due to missing data, bias in the measurement of outcomes, and bias in the selection of the reported result. The reviewers categorized the quality items as yes, probably yes, probably no or no. On the other hand, the risk of bias for the randomized clinical trial (RCT) was assessed in accordance to the Cochrane Reviewers’ handbook [20]) and consisted of the evaluation of six RCT-related items, namely: (i) random sequence generation, (ii) allocation concealment, (iii) the blinding of participants, personnel and outcome assessors (iv), the handling of incomplete outcome data, (v) selective outcome reporting and (vi) other sources of bias. All the included items were finally judged as adequate, inadequate or unclear (legend: + = adequate; ? = unclear; − = inadequate). Furthermore, the CONSORT guidelines for non-pharmacological treatments (NPT) were employed with the purpose of properly evaluating other sources of bias. To be more precise, the following issues were appraised: the setting of the study, therapist expertise, study design, statistical methods, calibration, sample size calculation, source of funding, smoking habit, and the necessity for hard or soft tissue improvement (legend: US = university setting, DTD = defined trial design, SH = smoking habit, SZC = sample size calculation, DSM = defined statistical methods, C = calibration, DF = disclosure of funding, HSTI = hard/soft tissue improvement, ET = experienced therapist). These tools were independently used by two reviewers (AP and DL) for the final assessment (k = 0.84).

**Table 1:** Summary of the features of the included studies (legend: n.m. = not mentioned; n.r. = not reported. Columns include the following data: first author, year of publication, follow-up, abutment material, interproximal papilla (PES), recession (REC) in mm, number of patients/sample size, gender, mean age/range, implant type, regenerative procedures, implant position, abutment type, implant survival rate, prosthetic survival rate, complications, pink esthetic score (Overall PES), midfacial mucosal recession (PES), drop-out and notes).

Study	Year	Follow-Up	Abutment Material	PES (Pink Esthetic Score) IntPapilla	REC	Number of Patients	Gender F/M	Mean Age/Range	Implant Type
Borges et al. [21] (CCT)	2014	12 months	cad-cam zirconia or cad cam grade 4 titanium coated with titanium nitride vs custom metal abutment in the control group	mean overall papilla score 1.66 +/− 0.48 relative to cad-cam group versus 1.05 +/− 0.65 relative to custom abutment group	n.m.	38	14 females and 24 males	49 years (range 28–90)	Osseospeed Astra Tech dental
Lops et al. [22] (CCT)	2014	24 months	stock zirconia or titanium (ZirDesign or TiDesign) abutment vs cad-cam zirconia or titanium abutment	n.m.	(marginalREC) 0.3 mm zirconia stock; 0.3 mm titanium stock; 0.1 mm zirconia cad-cam; −0.3 titanium cad-cam	72	33 females and 39 males	46 years (range 26–58)	Osseospeed Astra Tech dental
Borzangy et al. [23] (RCT)	2015	12 months	cad-cam zirconia vs stock titanium	baseline stock titanium 1.335 t12 stock titanium 1.535; baseline cad-cam zirconia 1.325 t12 cad-cam zirconia 1.415	Prefabricated abutments at 1 year and cad-cam customized abutments at 1 year showed improving papilla height about 0.5 mm (mean); on the contrary prefabricated abutment at 1 year showed less than 0.5 mm marginal recession and cad-cam customized abutments showed less than 0.5 mm (mean) coronal growth of the soft tissue margin	30	15 females and 14 males	45.03 ± 13.77 years (range 22–73)	Straumann
Schepke et al. [24] (RCT)	2017	12 months	cad-cam zirconia abutments vs stock (Zirdesign) abutment	t1 stock 1.0 t12 stock 1.6; t1 customized 1 t12 customized 1.7	n.m.	50	33 females and 17 males	48.3 years (range 18–79)	Osseospeed Astra Tech dental
Wittneben et al. [25] (multicenter RCT)	2017	12 months	stock zirconia abutment vs cad-cam zirconia abutment	baseline stock 1.395 t12 stock 1.415; baseline cad-cam 1.475 t12 cad-cam 1.475	n.m.	40	n.m.	n.m.	Bone level implant Straumann
Lops et al. [9] (CCT)	2017	24 months	stock zirconia or titanium (ZirDesign or TiDesign) abutment vs cad-cam zirconia or titanium abutment	n.m.	(intREC) 0.53 mm stock titanium; 0.52 mm stock zirconia; −0.46 custom zirconia; −0.56 custom titanium	72	33 females and 39 males	46 years (range 26–58)	Osseospeed Astra Tech dental
**Regenerative Procedures**		**Implant Position**		**Abutment Type**		**Implant Survival Rate**		**Prosthetic Survival Rate**	
12 out of 38 implants withresorbable membrane+ autologous bone		Maxilla between teeth 13–23		cad-cam, Atlantis; stock, CastDesign		100%		100%	
no hard and/or soft tissue augmentation procedure		from the second premolar forward		cad-cam, Atlantis; stock TiDesign or ZirDesign		98.60%		96%	
n.m.		single tooth gaps in the anterior maxilla position 14 to 24		Zr abutments (Etkon abutment, Straumann AG, Basel, Switzerland)/porcelain fused to Zr crowns or prefabricated anatomic Ti abutments (Straumann AG, Basel, Switzerland)/porcelain fused metal crowns were fabricated and delivered to a clinician		100% (authors reported implant success rate)		95.2% stock titanium abutment; 97.5% cad-cam Zirconia abutment	
n.m.		single mandibular or maxillary premolar		cad-cam Atlantis zirconia; stock ZirDesign zirconia		100%		100%	
contour augmentation		single tooth gaps in the anterior maxilla position 14 to 24		cad-cam Atlantis zirconia; stock ZirDesign zirconia		100%		94.7% stock abutment; 100% cad-cam abutment	
no hard and/or soft tissue augmentation procedure		from the second premolar forward		cad-cam, Atlantis; stock TiDesign or ZirDesign		98.60%		96%	
**Complications**		**Overall PES**		**Marginal Soft Tissue Stability**		**Drop-out**		**Note**	
n.r.		n.m.		n.m.		n.r.		none	
fracture of one implant in the cad-cam zirconia abutment group; 2 abutment unscrewing in the stock abutment group		n.m.		(marginalREC) 0.3 mm zirconia stock; 0.3 mm titanium stock; 0.1 mm zirconia cad-cam; −0.3 titanium cad-cam		n.r.		negative values indicate soft tissue growth	
Mechanical complications involved debonding of the crown form the abutment and abutment screw fracture. Debonding of the crown occurred twice in two different patients in the Ti group (2.38%) by the time of the one-month and one-year follow-up visits. Abutment screw fracture happened during the delivery of a Zr abutment (1.27%). Technical complications reported in this study were crown shade mismatch (1.19%) and veneering porcelain chipping (2.46%). Shade mismatch was noticed in the Ti group during initial delivery of the crown, and it was corrected before the final crown delivery. Minor veneer porcelain chipping occurred in one patient in each group at the six-month follow-up visit. One patient in the Ti group required just finishing and polishing to the crown to eliminate a sharp edge, while one patient in the Zr group received a small composite restoration.		prefabricated titanium abutments at 6 months = mean 8.31 (sd1.18); CAD/CAM customized zirconia abutments at 6 months = mean7.36 (sd1.80); Prefabricated titanium abutments at 1 year = mean 8.38 (sd1.19); CAD/CAM customized zirconia abutments at 1 year = mean 7.78 (sd1.93)		level of facial mucosa as item of PES: stock titanium t1 1.5; cad-cam zirconia t1 1.5; stock titanium t12 1.92; cad-cam zirconia 1.67		1		none	
n.r.		prefabricated zirconia abutments t1(two weeks) = 9.2 (1.8); cad-cam zirconia abutments t1(two weeks) = 9.0 (2.5); prefabricated zirconia abutment t12(twelve months) = 10.9 (1.6); cad-cam zirconia abutments t12(twelve months) = 10.6 (2.1)		level of soft tissue margin as item of PES: stock t1 1.4; stock t12 1.6; customized t1 1.5; customized t12 1.6		n.r.		none	
1 ceramic fracture (incisal edge) in the stock group		prefabricated zirconia abutment 1 year 7.00; cad-cam zirconia abutment 1 year 7.65;		level of labial mucosa as item of the modified PES: baseline stock 1.26; t12 stock 1.44; baseline cad-cam 1.65; t12 cad-cam 1.60		1		none	
fracture of one implant in the cad-cam zirconia abutment group; 2 abutment unscrewing in the stock abutment group		n.m.		n.m.		n.r.		negative values indicate soft tissue growth	

The descriptive statistics of the included studies were obtained by summarizing the main features of patients and implants for both groups. If at least two studies with comparable outcome variables were found, a meta-analysis was undertaken. The estimates of the effects of using the CAD/CAM versus prefabricated abutment were expressed as mean differences (MDs) for continuous variables, together with 95% confidence intervals. MDs were combined using a fixed-effects model (Mantel–Haenszel method), or a random-effects model, according to heterogeneity. If studies with different experimental designs could be included in the same meta-analysis, subgroup analysis was undertaken, by aggregating data from studies with the same design. the heterogeneity among studies was assessed using the Q Cochrane test (Chi2) and I2. Fixed effects meta-analysis was used when the heterogeneity was small (I2 < 60%, *p* > 0.05); otherwise, random-effects model analysis was undertaken. For the statistical evaluation, the patient was considered the unit of analysis. *p* = 0.05 was considered the significance threshold.

## 3. Results

**Figure 1:**
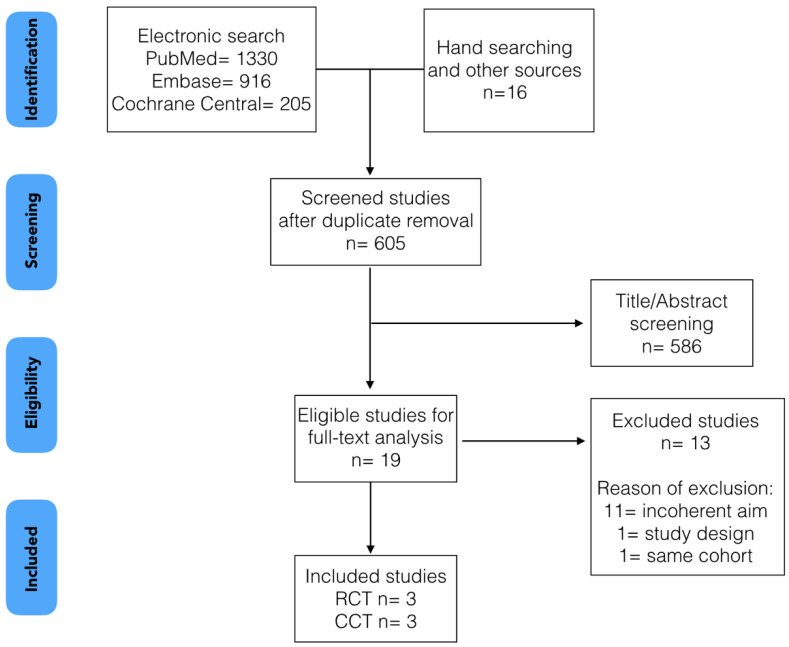
Flow diagram showing the screening process.

A flow diagram showing the screening process is reported in Figure 1. A total of 2467 studies were identified through the literature review: 1330 in MEDLINE/PubMed, 916 in EMBASE, 205 in the Cochrane Central Register of Controlled Trials (CENTRAL), and 16 through handsearching, crossreferencing and the gray literature search. After duplicate removal and title/abstract screening, 19 articles were selected for full-text analysis. Hence, 13 studies were excluded for the following reasons: the aim of the study was inconsistent with the aim of the systematic review [16,26,27,28,29,30,31,32,33,34,35], overlapping data/same cohort [36] and the study design was inconsistent with the inclusion criteria [37]. The reasons for exclusion are available in Supplementary Table S2. Following the application of inclusion and exclusion criteria, six articles were finally included: three randomized controlled clinical trials (RCTs) [23,24,25] and three controlled clinical trials (CCTs) [9,21,22], which underwent qualitative and quantitative analysis, respectively. The meta-analysis was performed on pink esthetic score (Overall PES), level of the midfacial mucosal recession (ML), which was a PES item (as described by Belser et al., 2009) [38], and interproximal papilla, which was calculated as the mean value of PES items for mesial and distal papilla.

### 3.1. Features of the Included Studies

The main features of the included studies are summarized in Table 1. RCTs were published between 2015 and 2017, while CCTs were published between 2014 and 2017. Among the selected studies, RCTs were approved by ethics committee, whereas as far as CCTs are concerned, it was not clearly specified. All studies were conducted in university settings, excepting for Borges et al., which was conducted in a private practice setting. Overall, 230 patients receiving 230 dental implants were treated in six studies (110 patients receiving 110 dental implants in CCTs and 120 patients receiving 120 dental implants in RCTs, respectively). The whole number of patients who dropped out (excluded or missing data) was six. More specifically, Wittneben et al. [25] reported that one patient passed away before treatment completion. Borges et al. [21] excluded one patient due to a missing contact point between the implant restoration and the adjacent teeth, while another patient was not reachable for the follow-up visit. Borzangy et al. [23] reported that two patients did not comply with follow-up visits, whereas one patient moved to another country. Clinical soft tissue data were reported a 12-month follow-up by Schepke et al. [24] and Borzangy et al. [23]; conversely, they were reported after 24 months by Lops et al. [9,22]. and 6, 12 and 36 months by Wittneben et al. [25,36] All clinical investigations referred to implant-supported single crown rehabilitations in the anterior maxillary region [21,23,25] or maxillary and mandibular regions [9,22,24], respectively. Dental implants were placed in the frontal area, meaning the segment between the second premolars. Globally, patients were enrolled and treated between January 2009 and February 2014. All inserted fixtures were bone-level dental implants obtained from two different companies (Astra Tech OsseoSpeed^®^ TX, Dentsply Sirona Implants, Göteborg/Mölndal, Sweden; or Straumann^®^ AG, Basel, Switzerland). Implant diameter and length ranged between 3.5 mm and 9–13 mm (Astra Tech OsseoSpeed^®^ TX, Dentsply Sirona Implants, Göteborg/Mölndal, Sweden) or 3.3–4.1 mm and 8–12 mm (Straumann^®^ AG, Basel, Switzerland), respectively. If requested, guided bone regeneration (GBR) procedures were performed in two studies at time of implant placement [21,25]. Four studies used both metal and zirconia abutments, while only zirconia abutments were used by Schepke et al., 2017 and Wittneben et al., 2017. Cemented single crowns were delivered by Borzangy et al. (2015) [23] and Lops et al. (2017) [9], while screw-retained single crowns were used by Wittneben et al. [25], Schepke et al. [24] and Borges et al. [21], respectively. Lops et al. [9,22], in two different studies based on the same patient sample, reported interproximal and marginal soft tissue recessions measured in millimeters. On the other hand, Borges et al. [21] reported soft tissue parameters as PES items: mesial papilla, distal papilla and interproximal papilla (mean value). The overall pink esthetic score (PES) and the score of every single item were reported in the three aforementioned RCTs.

### 3.2. Quality Assessment

The randomized controlled clinical trials (RCTs) were evaluated at unclear risk of bias (Table 2A), whereas the controlled clinical trials (CCTs) were judged at high risk of bias (Table 2B).

### 3.3. Heterogeneity Assessment

**Figure 2:**
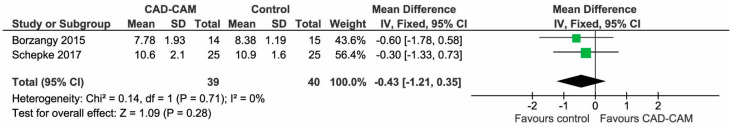
Forest plot of the overall pink esthetic score at 12 months. No statistically significant difference was found (MD −0.43; 95% CI −1.21, 0.35; *p* = 0.28) [23,24].

**Figure 3:**
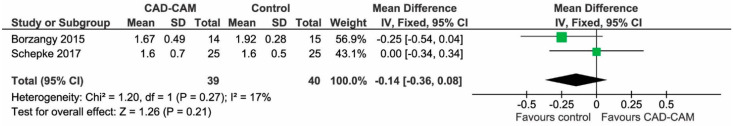
Forest plot of the marginal soft tissue level at 12 months. No statistically significant difference was found (MD −0.14; 95% CI −0.36, 0.08; *p* = 0.21) [23,24].

**Figure 4:**
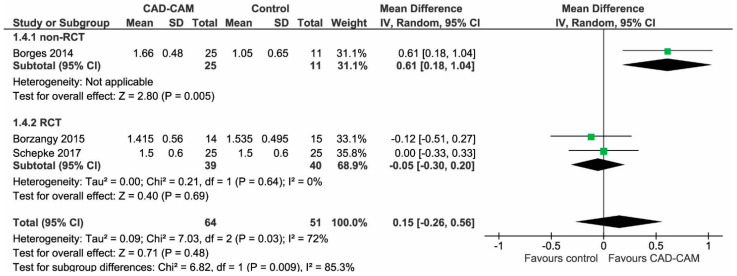
Forest plot of the value of interproximal papilla recession at 12 months. Studies were divided into two subgroups according to the study design. There was a significant difference in effect (*p* = 0.009), and significant heterogeneity (*p* = 0.03) between the two subgroups. Overall, no statistically significant difference was found in IntPapilla between using prefabricaded and customized CAD/CAM abutments (MD 0.15; 95% CI −0.26, 0.56; *p* = 0.48) [21,23,24].

No heterogeneity was observed for pink esthetic score (Overall PES, (Chi2: 0.14; df = 1 (*p* = 0.71)); I2 = 0%; Figure 2) and level of midfacial mucosal recession (ML) (Chi2: 1.20; df = 1 (*p* = 0.27); I2 = 17%; Figure 3). Conversely, heterogeneity was detected between subgroups for interproximal papillae recession (IntPapilla, (Chi2: 7.03; df = 2 (*p* = 0.03); I2: 72%); Figure 4).

### 3.4. Soft Tissue Stability

The meta-analysis was performed on 12-month data. No statistically significant differences were observed among abutment groups concerning either Overall PES (MD −0.43; 95% CI −1.21, 0.35; *p* = 0.28; Figure 2) or IntPapilla (MD 0.15; 95% CI −0.26, 0.56; *p* = 0.48; Figure 4), or in terms of level of midfacial mucosal recession (MD −0.14; 95% CI −0.36, 0.08; *p* = 0.21; Figure 3). For IntPapilla, a random effects meta-analysis was performed, due to heterogeneity between subgroups (*p* = 0.03). Interestingly, a significant difference in effects between studies with different designs was found for IntPapilla (Figure 4), suggesting that the experimental design could be a potential confounding factor that may affect the results. Since the risk of bias for non-RCT studies was high, unlike for the RCTs, it can be assumed that the latter provide more reliable results regarding the IntPapilla outcome.

## 4. Discussion

This systematic review and meta-analysis were performed in order to evaluate the role of CAD/CAM abutments on soft tissue response when compared to conventional stock abutments after at least 12 months of loading. The search strategy and the selection process led to the inclusion of six studies, namely, three RCTs [23,24,25] and three CCTs [9,21,22]. According to the available data, it can be stated that CAD/CAM abutments do not improve peri-implant marginal and/or interproximal soft tissue outcomes over the above-mentioned follow-up period. The present findings do not differ from those of previous systematic reviews, as no advantage was found for any type of abutment [6,18,19]. To the best of our knowledge this is the first systematic review and meta-analysis focusing on the relationship between abutment customization and soft tissue response. In fact, previous systematic reviews mainly evaluated different abutment materials and morphologies [18,39]. Thus, the effect of customization was analyzed only partially. In this regard, a systematic review and meta-analysis of CAD/CAM abutments was proposed by Raee et al. (2021) [19]. However, different aspects were analyzed, such as probing pocket depth (PPD), bleeding on probing (BOP), plaque index (PI), keratinized mucosal width (KMW) and pink aesthetic score (PES). It should be highlighted that the study conducted by Borges et al. (2014) [21] was excluded by Raee et al. (2021) [19], whereas it was included for qualitative and quantitative analysis in the current study. This was mainly due to the fact that Borges et al. (2014) [21] reported the value of mesial and distal papillae, which were evaluated as items in the pink aesthetic score (PES). Data extraction and calculation of the mean value allowed the statistician to run a meta-analysis. Hence, after a discussion, the authors decided to use the data in order to provide the best available evidence. Furthermore, no data were provided regarding the stability of marginal and interproximal soft tissues. Similarly, a meta-analysis was not conducted for the papilla height change by Zarauz et al. [6]. It is crucial to understand the behavior of soft tissue towards abutments to help select the most suitable solution. Stock abutments have been associated with unnatural shape of the mucosa between teeth and implants, poor aesthetics and difficulties in removing excess cement [40]. Computer-aided design (CAD) allows the clinician to shape and plan all features of morphology, including the emergence profile and angle of the restoration and their relationship with marginal and interproximal soft tissues [41]. This finding is worthy of note as emerging evidence revealed that the frequency of recessions is associated with the shape of the emergence profile [42]. Borges et al. compared zirconia and metal CAD/CAM abutments to metal stock abutments. After 12 months of function, better outcomes were observed for CAD/CAM abutments. These results were justified by the fact that CAD/CAM abutments provide ideal crown contours and peri-implant soft tissue support [21]. Among the included studies, Borges et al. [21] and Wittneben et al. [25] performed guided bone regeneration when needed and Lops et al. [9,22] reported no hard or soft tissue augmentation, whereas Borzangy et al. [23] and Schepke et al. [24] did not mention such circumstances. As a rule, GBR procedures influence hard and soft tissues around implants in a positive manner. Nevertheless, this might have no influence in the short-term follow-up. More interestingly, Borzangy et al. [23] and Wittneben et al. [25] reported no significant differences in terms of midfacial mucosal recession, whereas Lops et al. [9,22] reported statistically significant differences favoring customized CAD/CAM abutments. Indeed, a mean 0.3 mm recession was observed for both titanium and zirconia stock abutments after 2 years. Similarly, CAD/CAM abutments showed greater papilla fill, whereas a mean 0.53 mm papilla recession was observed in stock abutment groups [9]. Although titanium CAD/CAM abutments showed better outcomes in terms of soft tissue stability, it was emphasized that zirconia CAD/CAM abutments should be selected in thin mucosal phenotypes to prevent aesthetic impairment and grey appearance [22]. It has to be highlighted that measurements in two RCTs [23,25] were performed on cast models. Consequently, the effects of compression and distortion due to the impression material on soft tissues could not be excluded. As a consequence, it can be speculated that measuring methods that do not affect peri-implant soft tissues, such as photographic or digital model evaluation, might obtain different results. On the other hand, only Lops et al. [9,22] reported numeric values in millimeters, so direct comparison was not possible. The present meta-analyses showed no statistically significant differences when comparing CAD/CAM and stock abutments. This can be explained by the fact that the results refer to 12-month data. It can be hypothesized that the effect of soft tissue maturation may not be noticed. In this regard, several studies have demonstrated that papilla fill and improvement in marginal mucosal recession may still occur 2 years after the final prosthetic delivery [43]. Therefore, soft tissue outcomes should be evaluated when adequate stability has been achieved [43,44]. Another explanation as to why no clinical difference between stock and CAD/CAM abutments was found is related to the fact that abutment shape is not the only variable influencing soft tissue response. Indeed, soft tissues around implants in the aesthetic zone are affected by many factors, such as: initial mucosal phenotype, mucogingival procedures, the three-dimensional position of the fixture, features of the restoration and the periodontal attachment level of the neighboring teeth [24]. All these aspects may preclude drawing unequivocal conclusions. Numerous studies concluded that zirconia represents a more appropriate material for CAD/CAM abutment due to better color integration, epithelial and fibroblast adhesion, and lower plaque accumulation and inflammatory reaction [23]. Recent studies showed that zirconia can cause damage to the dental implant titanium interface under occlusal loading [45]. It should be considered that titanium degradation at the level of implant–abutment connection might lead to biologic and/or mechanical complications. Lack of stability and wear resistance at the interface between the abutment and the fixture could produce discrepancies greater than 10 microns. From a biological viewpoint, the increased space between components during function may promote microleakage with bacterial proliferation and percolation [46,47,48]. The wear and misfit at the implant–abutment interface was evaluated in a recent systematic review conducted by de Holanda et al. [49]. Interestingly, statistical differences were observed between titanium and zirconia abutments regarding the wear and misfit at the implant–abutment interface. The authors justified this finding, ascribing them to differences in the Young modulus. In fact, titanium abutments and fixtures have a similar Young modulus that is approximately 105 GPa. On the contrary, the Young modulus of zirconia is twice this value (approximately 210 GPa), leading to surface deformations at the implant–abutment interface. According to these considerations, vestibular and interproximal soft tissue stability might be influenced by connection, abutment type and material. Within the context of the present systematic review, Schepke et al. [24] and Wittneben et al. [25] employed only zirconia abutments, whereas the remaining studies applied both titanium and zirconia abutments. It can be speculated that misfit and microleakage may lead to mucosal recession in thin phenotypes and overgrowth associated with inflammation in cases of thick phenotypes. Nevertheless, due to the paucity of data, the meta-analysis could be conducted on 12-month data only. Indeed, among the included studies, only Lops [9,22] and Wittneben [36] reported follow-ups greater than 12 months. It should not be excluded that surface deformation at the implant–abutment interface has a clinical impact on peri-implant soft tissues. However, this condition might develop and deteriorate over years of function. Future studies including medium- to long-term RCTs comparing prefabricated (stock) versus customized abutment and reporting on the soft tissue outcomes should be conducted to further clarify the results of this systematic review and meta-analysis.

## 5. Strengths and Limitations

Some strengths of the present systematic review and meta-analysis were that three databases were searched and gray literature was included. This is relevant as there may be a trend towards publishing positive results, whereas negative outcomes are often not published. Hence, it is important to scrutinize gray literature in order to reduce the magnitude of reporting bias. Furthermore, only studies featuring appropriate methodologies (CCTs and RCTs) were analyzed. The heterogeneity of the included studies remains a substantial limitation. It is difficult to isolate the effect of customization as different materials (titanium and zirconia) and retention systems (screw- and cement-retained) were used. Another issue is related to the fact that two studies performed adjunctive guided bone regeneration (GBR) procedures, whereas the remaining studies placed dental implants in pristine bone or did not report such circumstances. The soft tissue response is influenced by a previous GBR procedure, usually with a more favorable result. Even though six studies fulfilled the inclusion criteria, due to a general lack of homogeneity concerning soft tissue evaluation and the follow-up periods, the meta-analyses were performed on only two or three studies. Consequently, few data could be included and analyzed. Although two studies may appear few, in the *Cochrane Handbook for Systematic Reviews of Interventions*, it is clearly stated that “meta-analysis is the statistical combination of results from two or more separate studies” [50]. In the meta-analysis on interdental papilla, studies with different experimental designs (two RCTs and one CCT) were included and separated in two subgroups. While the two RCTs showed similar effects, a significant difference between subgroups was found, suggesting that the study design might play a role in determining the outcome. For all these reasons, the results of this review should be interpreted with caution.

## 6. Conclusions

- Within the limitations of the present systematic review and meta-analysis, at the time of writing, the evidence does not favor customized CAD/CAM abutments when compared to prefabricated abutment in the restoration of single edentulism in the aesthetic zone after 12 months of loading. Due to their peculiarities, it cannot be excluded that CAD/CAM abutments improve soft tissue support and stability around implants. However, the effect of soft tissue maturation over time could not be revealed due to the limited follow-up periods of the available data.
- Future studies should consider soft tissue evaluation and related measurements by means of standardized methods. Marginal and interproximal soft tissues should be evaluated both qualitatively (PES) and quantitatively (numeric measurements, millimeters).
- Due to increased cost and time, the usage of customized CAD/CAM abutments in everyday clinical practice should be based on a careful case-by-case evaluation.
- Since studies characterized by adequate methodology and follow-up are lacking, further research is warranted.

## Figures and Tables

**Table 2:** (**A**) Quality assessment of RCTs performed in accordance with the *Cochrane Handbook for Systematic Reviews of Interventions* (legend: + = adequate; ? = unclear; − = inadequate; US = university setting, DTD = defined trial design, SH = smoking habit, SZC = sample size calculation, DSM = defined statistical methods, C = calibration, DF = disclosure of funding, HSTI = hard/soft tissue improvement, ET = experienced therapist). (**B**) quality assessment of CCTs performed through ROBINS-I.

**Study**	**Random Sequence Generation**	**Allocation Concealment**	**Blinding of Assessor**	**Blinding of Statistician**	**Incomplete Outcome Data**	**Other Sources of Bias**	
Borzangy et al. [23] 2015	?	+	+	?	+	US, DTD, SH, DSM, C	
Schepke et al. [24] 2017	+	+	−	+	+	US, DTD, DSM, SZC, DF	
Wittneben et al. [25] 2017	?	+	+	?	?	US, DTD, ET, DSM, HSTI, DF	
**Study**	**Bias due to Confounding**	**Bias in Selection of Participants into the Study**	**Bias in Classification of Interventions**	**Bias due to Deviations from Intended Interventions**	**Bias due to Missing Data**	**Bias in Measurement of Outcomes**	**Bias in Selection of the Reported Result**
Borges et al. [21] 2014	Probably no	No	No	No	Probably no	Probably yes	No
Lops et al. [22] 2015	Moderate risk	Low risk	Low risk	Low risk	Low risk	Moderate risk	Low risk
Lops et al. [9] 2017	Probably yes	No	No	No	No	Probably no	No

## Data Availability

The extraction forms, figures, tables and data synthesis are available upon request to antonino.palazzolo@unimi.it.

## Supplementary Materials

The following supporting information can be downloaded at: https://www.mdpi.com/article/10.3390/jcm12113847/s1, Table S1: Databases and search items; Table S2: Reasons of exclusions.
