# Supplementary material for: CAD/CAM Abutments in the Esthetic Zone: A Systematic Review and Meta-Analysis of Soft Tissue Stability
Source: J Clin Med. 2023 Jun 4;12(11):3847. doi: 10.3390/jcm12113847 (PMC10253332; doi:10.3390/jcm12113847)
Supplement: Supplementary file 1 [file jcm-12-03847-s001.zip › jcm-2352604-supplementary.pdf]

Supplementary Table S1: Databases and search items.

Databases	Keywords
**PubMed**	(anterior OR esthetic) AND (abutment OR dental implant OR zirconia OR titanium abutment OR CAD/CAM abutment OR custom abutment) AND (gingival margin OR papilla OR soft tissue stability OR gingival recession)
**Embase**	((anterior OR esthetic) AND ('implant'/exp OR implant) AND abutment OR 'titanium'/exp OR titanium OR 'zirconium oxide'/exp OR 'zirconium oxide') AND ('dental'/exp OR dental) AND ('implant'/exp OR implant) AND ('computer aided design/computer aided manufacturing'/exp OR 'computer aided design/computer aided manufacturing')
**Cochrane Central Register of Controlled Trials**	(((dental AND implants OR (dental AND abutment) OR (dental AND 'implant abutment' AND design) OR (dental AND prosthesis, AND 'implant supported')) AND 'computer aided' AND design OR ('computer assisted' AND design) OR ('computer aided' AND manufacturing) OR 'cad cam' OR (zirconia AND abutment) OR (zirconia AND dental AND abutment) OR (custom AND abutment)) AND stock AND abutment OR (titanium AND abutment) OR (zirconia AND abutment)) AND pink AND esthetic AND score OR papilla OR (soft AND tissue AND stability) OR (gingival AND recession) OR ('peri implant' AND soft AND tissue)

Supplementary Table S2: Reasons of exclusions.

Study	Reason of exclusion
Joda et al. [26] 2015	the aim of the study was incoherent with the aim of the Systematic review
Joda et al. [27] 2015	the aim of the study was incoherent with the aim of the Systematic review
Joda et al. [28] 2016	the aim of the study was incoherent with the aim of the Systematic review
Joda et al. [29] 2016	the aim of the study was incoherent with the aim of the Systematic review
Wittneben et al. [36] 2020	overlapping data, same cohort of Wittneben 2017
Costa et al. [30] 2021	the aim of the study was incoherent with the aim of the Systematic review
Rathe et al. [31] 2021	the aim of the study was incoherent with the aim of the Systematic review
Fonseca et al. [37] 2021	study design inconsistent with inclusion criteria
Donker et al. [32] 2022	the aim of the study was incoherent with the aim of the Systematic review
Wehner et al. [33] 2022	the aim of the study was incoherent with the aim of the Systematic review
Hsu et al. [34] 2022	the aim of the study was incoherent with the aim of the Systematic review
Lin et al. [16] 2022	the aim of the study was incoherent with the aim of the Systematic review
Tartea et al. [35] 2023	the aim of the study was incoherent with the aim of the Systematic review
